# Pathways to decoding the clinical potential of stress response FOXO-interaction networks for Huntington's disease: of gene prioritization and context dependence

**DOI:** 10.3389/fnagi.2013.00022

**Published:** 2013-06-13

**Authors:** Frédéric Parmentier, François-Xavier Lejeune, Christian Neri

**Affiliations:** Laboratory of Neuronal Cell biology and Pathology, INSERM Unit 894, CNRS UMR 7102, University Pierre and Marie CurieParis, France

**Keywords:** Huntington's disease, cellular-stress response, FOXO network, clinical potential, gene prioritization, system-level approach, entropy

## Abstract

The FOXO family of transcription factors is central to the regulation of organismal longevity and cellular survival. Several studies have indicated that FOXO factors lie at the center of a complex network of upstream pathways, cofactors and downstream targets (FOXO-interaction networks), which may have developmental and post-developmental roles in the regulation of chronic-stress response in normal and diseased cells. Noticeably, FOXO factors are important for the regulation of proteotoxicity and neuron survival in several models of neurodegenerative disease, suggesting that FOXO-interaction networks may have therapeutic potential. However, the status of FOXO-interaction networks in neurodegenerative disease remains largely unknown. Systems modeling is anticipated to provide a comprehensive assessment of this question. In particular, interrogating the context-dependent variability of FOXO-interaction networks could predict the clinical potential of cellular-stress response genes and aging regulators for tackling brain and peripheral pathology in neurodegenerative disease. Using published transcriptomic data obtained from murine models of Huntington's disease (HD) and post-mortem brains, blood samples and induced-pluripotent-stem cells from HD carriers as a case example, this review briefly highlights how the biological status and clinical potential of FOXO-interaction networks for HD may be decoded by developing network and entropy based feature selection across heterogeneous datasets.

## Introduction

The FOXO family of transcription factors is well-known for its effect in regulating longevity as initially uncovered in the nematode *C. elegans* (Kenyon et al., 1993). This activity of FOXO proteins may hold true in humans since allelic variation in FOXO3A was associated with the ability to be long-lived in several populations of centenarians (Willcox et al., 2008; Anselmi et al., 2009; Flachsbart et al., 2009; Li et al., 2009; Soerensen et al., 2010; Kleindorp et al., 2011). Besides their role as longevity-promoting factors, FOXO proteins are known to regulate a variety of biological processes which are important for development, metabolism and tumor suppression (Calnan and Brunet, 2008). Multiple studies have indicated that FOXO factors may lie at the center of a complex network of upstream pathways such as the PI3K-AKT (insulin/IGF-1 signaling cascade), MST-1, JNK, SIR-2, and AMPK pathways, cofactors such as 14-3-3 proteins and ß-catenin and a fairly large number of either established or putative transcriptional targets. This notion has been extensively reviewed in several articles to which to refer for more details (Greer and Brunet, 2005; Calnan and Brunet, 2008; Landis and Murphy, 2010; Yen et al., 2011; Neri, 2012; Eijkelenboom and Burgering, 2013). These studies have emphasized a model in which, through a series of context-dependent post-translational modifications and nucleo-cytoplasmic interactions, FOXO proteins are signal integrators that may be repressed by insulin/IGF-1 signaling and that may function developmentally or post-developmentally to modulate cell cycle arrest, apoptosis, autophagy, angiogenesis, differentiation, stress resistance, stem cell maintenance, glucogenesis, and food intake.

Consistent with their role in protecting from chronic-stress in a variety of cellular contexts, FOXO factors and interactors such as *sir-2.1*/SIRT1 and ß-catenin also regulate cellular proteotoxicity and neuron survival in models for neurodegenerative diseases such as Huntington's disease (HD) (Morley et al., 2002; Parker et al., 2005, 2012; Burnett et al., 2011) and Alzheimer's disease (AD) (Cohen et al., 2006; Kim et al., 2007). The same notion has been exemplified in more generic models of neurodegeneration (Calixto et al., 2012) and models for muscle cell dysfunction in oculo-pharyngeal muscular dystrophy (Catoire et al., 2008; Pasco et al., 2010). Interestingly, the protective effect of reducing the insulin/IGF-1 signaling cascade—which activates FOXO, is conserved from *C. elegans* to mammals (Cohen et al., 2009; Freude et al., 2009; Killick et al., 2009). FOXO proteins are not the sole proteins that may regulate cellular proteotoxicity by acting downstream to the insulin/IGF-1 signaling cascade as other transcription factors such as the heat shock factor HSF-1 may also be involved (Cohen et al., 2010; Teixeira-Castro et al., 2011; Zhang et al., 2011; Chiang et al., 2012). Collectively, these observations suggest that the activity of stress-response networks such as FOXO-interaction networks could modify the speed at which the pathogenic process develops in neurodegenerative disease. The FOXO-interaction networks contain several genes that are both potential drug targets (Russ and Lampel, 2005) and evolutionary-conserved, which provides a positive framework for investigating whether these networks might contain targets and markers of interest to tackle neurodegenerative diseases such as HD and AD.

## Pathways to decoding the clinical value of FOXO-interaction networks

Given the importance of FOXO-interaction networks for the regulation of cellular homeostasis, understanding the cellular, mechanistic and time requirements for these networks to regulate diseased neuron resistance and, possibly, modify the onset and progression of neurodegenerative disease has great therapeutic implications. Insight into this question may be provided by the unbiased analysis of stress-response network activity. One challenge is to identify the FOXO targets that may be involved in neuronal resistance in specific neurodegenerative disease conditions. Another challenge is to define the clinical potential of this information, which may be achieved by means of candidate gene prioritization. The knowledge required to prioritize genes in stress-response networks can be found in molecular profile datasets such as transcriptomic data. As molecular profile datasets are becoming increasingly available for studying neurodegenerative disease, a timely question is whether there is some supporting evidence for specific genes in FOXO-interaction networks to regulate the pathogenic process in neurodegenerative disease. A related question is whether some of these genes could be viewed as “privileged disease targets” whereas other genes might be viewed as “privileged predictors” of disease onset and progression. Here, the analysis of HD datasets may provide some answers.

### Huntington's disease datasets

HD is a dominantly-inherited disease with CAG expansion in the huntingtin (htt) gene and expanded polyglutamine (polyQ) tracts in the htt protein causing striatal and cortical degeneration (Walker, 2007). While HD is inherited, this disease shows a great deal of phenotypic variability and has become a subject of intense research to understand neurodegenerative disease biology due to genetic tractability, large number of models across species, shared disease mechanisms between HD and other neurodegenerative diseases (Zuccato et al., 2010) and availability of well-characterized cohorts of HD subjects (Orth et al., 2011). Among the many genes that could be targeted in HD (Zuccato et al., 2010), those genes which belong to stress response networks are of high interest as they regulate survival mechanisms that may be central to diseased-neuron resistance. Stress response networks encompass a large number of pathways that have been interrogated in genome-wide studies. Most particularly, transcriptomic data have been generated from (1) the striatum of several murine models of HD such as N-terminal htt transgenic mice R6/2 (at 6 and 12 weeks) and D9-N171-98Q (a.k.a. DE5; at 14 months) (Kuhn et al., 2007; Thomas et al., 2011), full length htt transgenic mice YAC128 (at 12 and 24 months) and knock-in mice CHL2 (at 22 months) and HdH(Q92/Q92) (at 18 months) and (2) caudate nucleus and BA4/BA9 cortex from post-mortem HD brains (Hodges et al., 2006), blood samples from pre-symptomatic and symptomatic HD carriers (Borovecki et al., 2005) and HD induced pluripotent stem (iPS) cells that were differentiated into neural stem cell (NSC) lines and that expressed 60 or 180 CAG repeats (2012). This represents a total of 14 HD contexts (seven murine and seven human contexts), all of them assessed on Affymetrix platforms. It is important to note that these 14 HD-related studies are heterogeneous in terms of htt gene species, genetic background, cellular/tissular context and pathological stage (Table S1). Additionally, there might be some level of cross-studies variability. Nonetheless, these data provide a case example to illustrate how the status and properties of FOXO-interaction networks might be explored in HD in the context of heterogeneous datasets.

### FOXO-interaction networks

**Figure 1:**
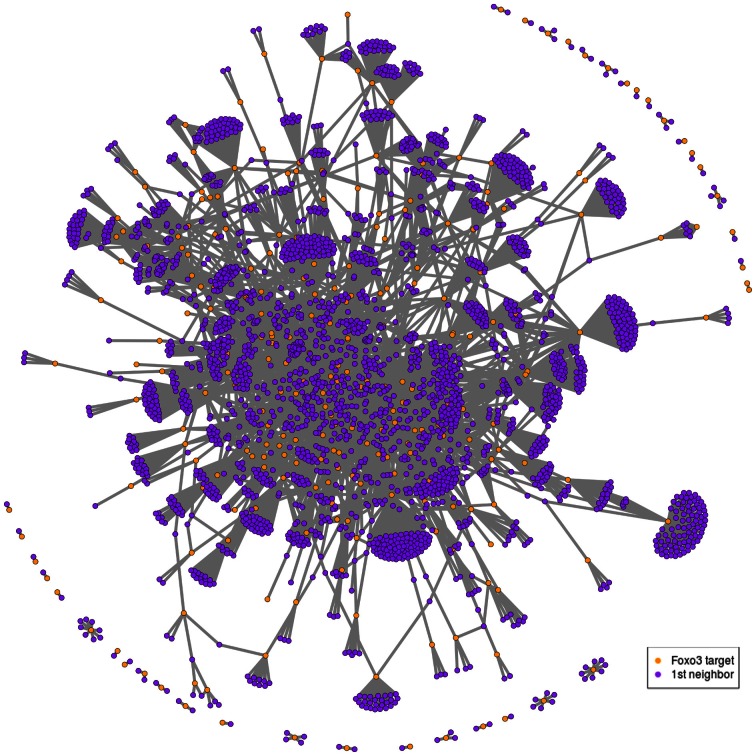
**Example of FOXO3-interaction network. Putative FOXO3 targets (orange nodes) as indicated by studies in mouse NSCs (Paik et al., 2009; Renault et al., 2009) with their first-degree neighbors (violet nodes) as inferred from the probabilistic functional network STRING.** This corresponds to 255 FOXO3 targets that have 2689 first neighbors in STRING (confidence score greater than 0.7) out of the 374 putative targets initially identified by the Paik et al. and Renault et al. studies. The confidence score was set at 0.7 to ensure clarity of the graph. Biological content is illustrated in Figure S1.

Putative targets of mammalian FOXO, namely FOXO3, have been identified in mouse NSCs (Paik et al., 2009; Renault et al., 2009; Ro et al., 2012), which represents a total of 374 genes emphasized by either study. As inferred from the probabilistic functional network STRING (Franceschini et al., 2013), 350 out of 374 mouse FOXO3 targets have a total of 5859 high-confidence (STRING score > 0.4) and first-degree interactors. This analysis results in a FOXO3-interaction network that contains 6209 genes (Figures 1, S1; Table S2) and that shows a large proportion of evolutionary-conserved genes. This network has small-world network characteristics (network in which most genes are not neighbors of one another, but most genes can be reached from every other by a small number of interactions), which may reflect the existence of “functional units” in this network. Several FOXO3 target groups are represented in this network, comprising, for example, genes involved in mTor signaling, p53 signaling, metabolic pathways, glycolysis, regulation of actin skeleton, cancer pathways, focal adhesion and cell cycle (Figure S1).

Intersecting this information with the HD microarray data abovementioned and selecting the genes which are instructed by at least 6 contexts within species and 11 contexts across species retains 4436 genes from murine datasets, 4881 genes from human datasets—here the best reciprocal hits as indicated by Ensembl (http://www.ensembl.org/), and 4634 genes from both murine and human datasets. What may be the behavior of these genes across HD contexts? This question can be addressed by using entropy based feature selection.

### Entropy based feature selection

**Figure 2:**
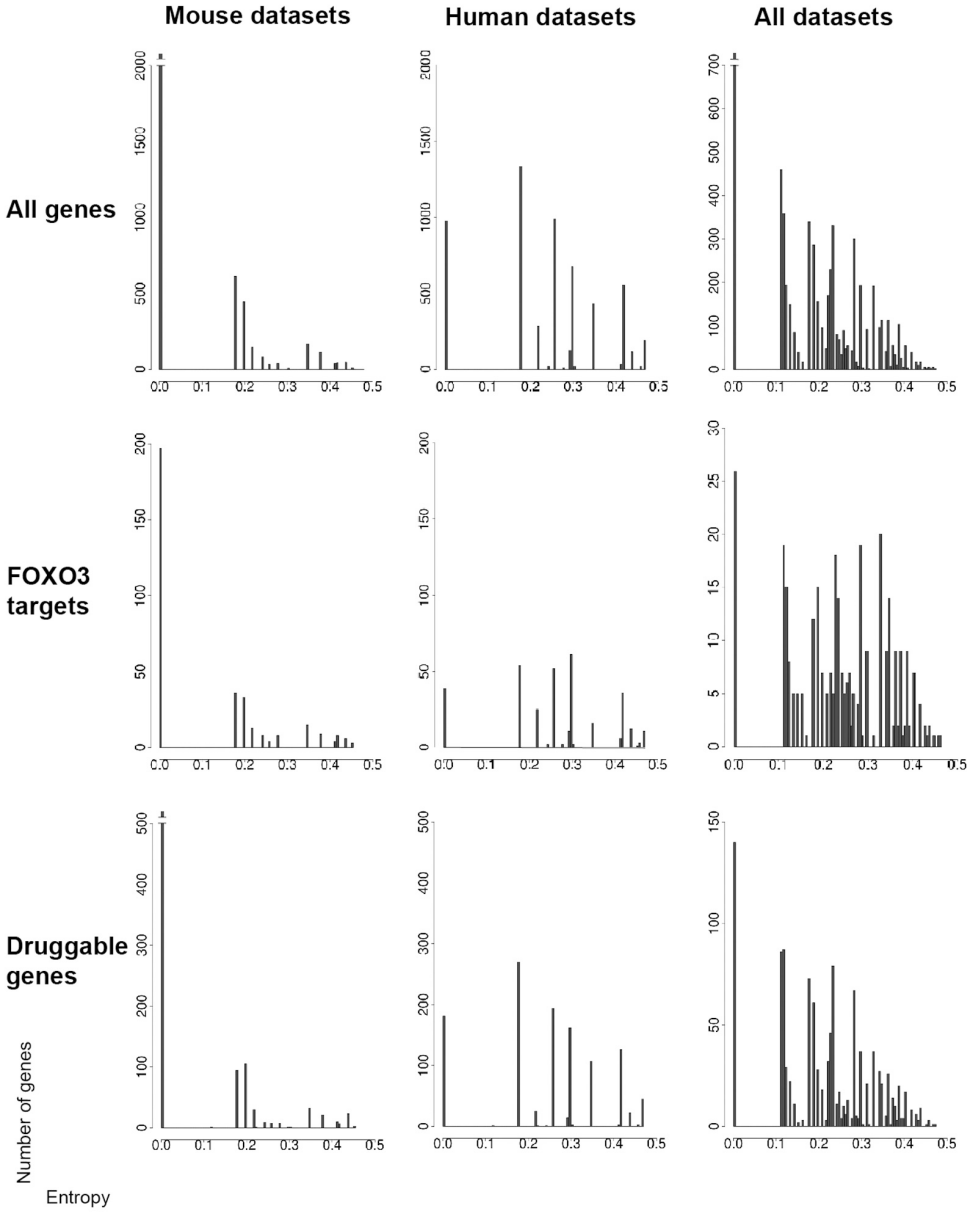
**Gene entropy values across murine and human datasets.** Datasets (seven conditions in the mouse and seven conditions in humans) are listed in the main text. Gene entropy values are shown for three categories of genes (all genes, FOXO3 targets only, druggable genes only) and for genes which are (1) retained by intersecting transcriptomic and gene-interaction data and (2) further instructed by at least six conditions in the mouse, six conditions in humans and 11 conditions in both species. The maximally achievable entropy value in these analyzes is 0.5. The corresponding gene lists are shown in Tables S3–5 as organized by species or across species and as shown for all numbers of instructive conditions.

Entropy is a mathematically defined quantity that helps to account for the flow of information through a biologically regulated process, and this quantity can be defined for individual genes as inferred from the change in status or level of activity across a number of experimental contexts. High gene-entropy values indicate context-dependent activity, and, conversely, low gene-entropy values indicate context-independent activity. In other words, entropy—as per mRNA-level standard, is a measure of how dependent is the variation of gene expression level on experimental context. In so far, entropy allows detecting the genes that may be particularly important for adaptability and homeostasis across cell types and across time. Regarding HD datasets (Table S1), entropy allows detecting the genes that may be particularly important for adaptability and response to mutant htt expression in specific cell types and species, and as pathology develops. A simple entropy analysis (Fuhrman et al., 2000) of HD datasets in which the signal is the change of gene status among three possibilities (up-regulated, down-regulated, no effect) allows genes with low-to-high entropy values to be identified in FOXO-interaction networks (Figure 2; Tables S3–5), which is also true when considering gene subsets such as FOXO3 targets (Paik et al., 2009; Renault et al., 2009; Ro et al., 2012) and potential drug targets (Russ and Lampel, 2005). Permutation analysis and statistical comparisons of gene entropy distributions before and after permutations (Mielke and Berry, 2007) suggests that these observations did not occur by chance (Figure S2). In these networks, FOXO1 and FOXO3 showed moderate to high entropy values across datasets (except, however, for FOXO3 across human datasets). The sirtuin SIRT1 showed high entropy values and sirtuin SIRT3 showed moderate entropy values across the murine and murine/human datasets (Tables S3–5). These observations are consistent with the notion that SIRTs such as SIRT1 and FOXOs such as FOXO3 may be highly sensitive to the context (cell type, species) in which they operate and they support the previously-emphasized importance of these genes in regulating mutant htt cytotoxicity (Parker et al., 2005, 2012; Jeong et al., 2011; Jiang et al., 2011; Fu et al., 2012).

Overall, it appears that the numbers of very high entropy (>0.4) genes are much smaller compared to that of low-to-moderate entropy genes, a trend notably illustrated by gene-entropy distributions across the 14 mouse and human contexts (Figure 2). This suggests that a rather limited proportion of genes in FOXO3-interaction networks might be highly sensitive to change in cellular context. While this might be unexpected considering that FOXO pathways are believed to be highly context-dependent, the genes that have middle to high entropy values (>0.2) are in larger numbers and may also be involved in context dependency. Additionally, the signal analyzed herein is based on the change of gene status, and not the amplitude of this change, which may limit the sensitivity of the analysis.

**Figure 3:**
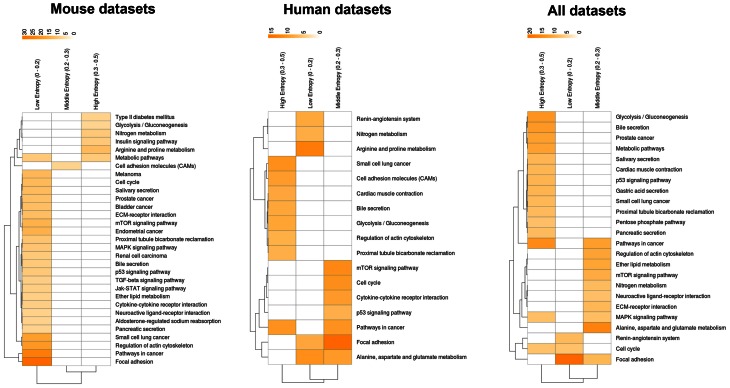
**Heat-maps illustrating the biological content of FOXO3 target genes as a function of gene entropy values.** Biological content is inferred using enrichment in KEGG pathways (*P* < 0.001) for three (arbitrarily selected) entropy categories corresponding to low, middle and high entropy values. The color code indicates −log *P*-values.

**Figure 4:**
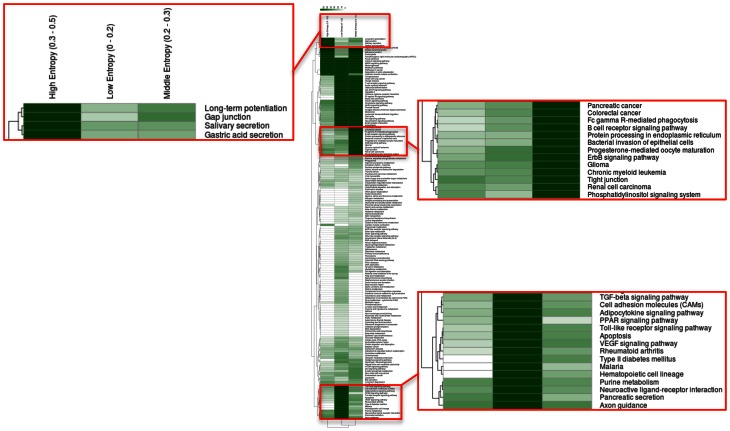
**Heat-maps illustrating the biological content of FOXO3-interaction networks as a function of gene entropy values.** Biological content is inferred using enrichment in KEGG pathways (*P* < 0.001) for three (arbitrarily selected) entropy categories corresponding to low, middle, and high entropy values. Details of the full heat-maps can be visualized in Figures S3–5. The color code indicates −log *P*-values. Gene contents are shown in Tables S6–8.

How about the biological content of gene-entropy categories? Enrichment analysis using KEGG annotations (Kanehisa et al., 2002) suggests that low-to-high entropy gene categories significantly differ in biological content, a phenomenon that is true for FOXO3 targets as well as larger gene sets and that is observed within and across species (Figures 3, 4). More precisely, gene-entropy categories may have specific KEGG-pathway profiles, and a given gene-entropy category may change biological profile across models of HD, providing a comprehensive view on how cells and tissues might respond to mutant htt expression within and across species. For example, regarding FOXO3 targets (Figure 3; Tables S6–8), the pathway “regulation of actin cytoskeleton” is specific to low-entropy genes in murine striatum datasets whereas it is specific to high-entropy genes in human datasets. This suggests that whereas this pathway may poorly respond to variable contexts such as htt gene species in HD mice, it may greatly respond to variable contexts such as cellular context (e.g., caudate nucleus, cortex, blood, and iPS cells) as contributed by the human datasets. Similar clear-cut evidences are provided by the KEGG-pathway profiles when considering FOXO3 targets and their high confidence first-degree neighbors (Figures 4, S3–5; Tables S9–11). For example, the annotation of gene-entropy categories corresponding to all datasets (14 conditions) highlights pathways that are more specifically linked to low, middle or high entropy (Figures 4, S6), providing a global signature for the biological significance of gene entropy categories. Interestingly, this signature dramatically changes when considering either murine or human datasets only (Figures S4, S5; Tables S10, S11), illustrating how FOXO3-interaction networks may be sensitive to the HD context(s) in which they operate.

**Figure 5:**
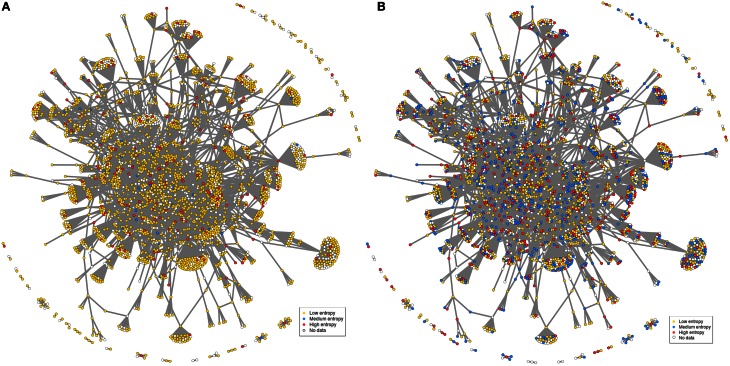
**Context-dependent distribution of gene entropy values in FOXO3-interaction networks.** The network used in this figure is the same as in Figure 1, except for labels of gene nodes. The categories for gene entropy used in this figure are the same as those in Figure 2. **(A)** Distribution of gene entropy values in the context of seven murine datasets. **(B)** Distribution of gene entropy values in the context of all datasets (addition of seven human datasets).

How is this translated at the gene level? If considering a rather homogeneous group of conditions, here murine striatum datasets, it appears that the corresponding FOXO3-interaction network mostly contain low entropy genes, suggesting that few genes only (medium to high entropy genes) such as for example FOXO1 (Table S6) may respond to change of context (e.g., htt gene species, genetic background, pathological stage) across murine models (Figure 5A). In contrast, many of the previously low-entropy genes such as for example the PTEN and DUSP6 phosphatases (Tables S6, S8) become middle-to-high entropy genes when adding the context variability associated to the human datasets (Figure 5B). This illustrates how the response of FOXO3-interaction networks to a change of context across HD-related conditions may be precisely mapped at the gene level (see also Figure S6). In summary, the examples provided herein illustrate how entropy based feature selection may allow stress response genes to be finely prioritized in HD. Of note, these examples are biased toward a FOXO3-interaction network that was selected “by hand” for the purpose of illustration. As such, they do not constitute final results nor they support final conclusions.

### Gene prioritization using entropy based feature selection

Many pathways may be closely related to the pathogenesis of neurodegenerative diseases such as HD. While hypothesis-driven approaches may allow the pathological or protective role of these pathways to be predicted, it remains unclear how these pathways may behave across multiple experimental models, whether they may be prominently associated to specific contexts and how this may impact on gene prioritization. The approach illustrated herein provides a glimpse of this problem. In particular, this approach roughly highlights two categories of genes including genes with a rather limited (low entropy genes or “stable genes”) or significant (moderate to high entropy genes or “unstable genes”) change of status across HD conditions. What is the significance of gene-entropy categories for prioritizing candidate disease targets? Whereas one may propose to employ stable genes as “privileged candidate targets” since the way to manipulate their activity in order to protect from the disease would make little doubt, other may consider that stable genes are targets of poor interest because they are unlikely to reflect the changes that may occur in specific disease contexts (e.g., cellular contexts, time requirements). These two possibilities are two perspectives on a single property, namely, the ability to change status across contexts. The negative perspective highlights insufficient selectivity in responding to a particular context, which might become practically useless in discovery efforts aiming at targeting specific aspects of the disease process. However, low entropy across heterogeneous datasets might constitute a preferred criterion for prioritizing candidate biomarkers. The more positive perspective may be based on the consideration that focusing on stable genes may allow a quick identification of “targets” with sufficient information whose therapeutic value can be established in subsequent target validation steps. In the context of the currently available HD datasets, all of them greatly differing at several levels such as htt gene type, genetic background, cellular context and disease stage, our bias is that genes having moderate to high entropy values may represent candidate targets of higher interest because manipulating their activity could more significantly impact on the homeostasis and survival of specific cell types at a given phase (e.g., early vs. late stage) and site (e.g., brain/neuronal vs. peripheral pathology) of the pathogenic process in HD. Considering additional parameters in the target gene prioritization model such as local network entropy, proximity to htt, brain gene expression and time requirement will refine the view on this question.

An important aspect of entropy based systems modeling is the context of the experiments used for gene prioritization. As illustrated herein, the nature and diversity of HD-related conditions may impact on the gene content and biological profiles of signal entropy categories. Specific experimental models may be responsible for most of the entropy observed (herein, the models based on human samples and cells), especially if the number of models is rather limited, which gains to be accounted. More largely, using comparable rather than divergent models of disease may change the interpretation of low-to-high entropy values in term of clinical potential and the final selection of genes as either promising targets or markers, or both. In theory, if analyzing multiple studies all examining for the same context (e.g., same cellular context at the same stage of pathology vs. control), then high entropy could indicate that a gene is unreliable, and middle-to-low entropy might be viewed as a robust criterion for prioritizing candidate targets. In practice, this type of situation is unlikely to be frequently encountered. A major trend for understanding context dependency in HD is to examine for the effects of multiple variables such as expanded-polyQ length, cellular context and pathological stage in individual species and models, then to select variables of interest and compare for their effects on gene behavior across species and models. In so far, context heterogeneity is a constitutive element of the framework for modeling HD datasets. High entropy is then anticipated to be relevant to several situations in which the comparison of the molecular and biological features that may be recapitulated by individual experimental models (model sensitivity analysis) and the subsequent selection of targets of interest relative to multiple contexts and variables (target prioritization) are two complementary aspects of systems modeling for HD.

## Perspectives

System-level approaches are key to target and marker discovery because these approaches are able to summarize and prioritize the biological information buried in large and complex datasets. Applying system-level approaches to the study of neurodegenerative diseases such as HD is an emerging approach that is anticipated to become widely used in the field as more diverse genome-wide datasets. Although developing system-level approaches requires specific skills in software computing and mathematics, databases and knowledge discovery platforms may become available and facilitate the access to these approaches.

There is an increasingly large repertoire of methods for the interrogation of complex datasets and development of systems modeling in disease research, ranging from bionetwork mapping (Rapaport et al., 2007; Lejeune et al., 2012) to reverse engineering of gene networks (Lefebvre et al., 2012) and analysis of network rewiring response and differential entropy properties (Bandyopadhyay et al., 2010; Califano, 2011; Shou et al., 2011; West et al., 2012). Regardless of how disease-associated networks are generated, understanding how these networks may differ and how they may reconfigure activity as a function of the context in which they operate is essential to gene prioritization. In this respect, entropy-based approaches may capture gene essentiality changes across multiple conditions that encompass several species and models. This is particularly attractive for studying the dynamics of chronic-stress response networks that, similarly to FOXO-interaction networks, may be strongly dependent on the context in which they operate. Entropy based feature selection may indeed help understanding how chronic-stress response networks allow specific cell types to maintain function and resist degeneration, and how cellular resistance may develop over time. This knowledge may in turn help unraveling the clinical potential of these networks for neurodegenerative diseases such as HD, which might foster the identification of successful disease-modifying strategies.

It is remarkable that longevity-promoting factors such as FOXO proteins may have a role in development and phenotypic plasticity (De La Torre-Ubieta et al., 2010; Christensen et al., 2011; Tang et al., 2011; Mei et al., 2012; Salih et al., 2012). FOXO proteins may thus be important throughout the entire lifetime of an individual. This raises the possibility that FOXO proteins regulate developmental, post-developmental and late determinants of the pathogenic process in HD. Today, data are relatively scarce to study context dependency in neurodegenerative disease. However, molecular profile data allowing context dependency to be examined in a deeper manner are becoming increasingly available, and their analysis using systems modeling is expected to tell us more about the clinical potential of stress response genes in HD and, perhaps, other neurodegenerative diseases.

### Conflict of interest statement

The authors declare that the research was conducted in the absence of any commercial or financial relationships that could be construed as a potential conflict of interest.

## Supplementary material

Table S1 **Fourteen models of HD pathogenesis for which transcriptomic data are available**.

Table S2 **List of 6209 mouse genes including FOXO3 targets and first-degree neighbors**.

Table S3 **Entropy of FOXO3-interaction-network genes across seven HD mouse datasets**.

Table S4 **Entropy of FOXO3-interaction-network genes across seven human HD datasets**.

Table S5 **Entropy of FOXO3-interaction-network genes across 14 HD-mouse and human-HD datasets**.

Table S6 **Annotation of gene-entropy categories for FOXO3 targets across murine datasets**.

Table S7 **Annotation of gene-entropy categories for FOXO3 targets across human datasets**.

Table S8 **Annotation of gene-entropy categories for FOXO3 targets across all datasets**.

Table S9 **Annotation of gene-entropy categories for the entire FOXO3-interaction network across murine datasets**.

Table S10 **Annotation of gene-entropy categories for the entire FOXO3-interaction network across human datasets**.

Table S11 **Annotation of gene-entropy categories for entire FOXO3-interaction network across all datasets**.

Figure S1 **Heat-map representing the biological content for 350 mouse FOXO3 targets and their 5859 first neighbors (STRING confidence score greater than 0.4).** Biological annotations are inferred from KEGG pathways. The cut-off for enrichment in KEGG pathways is *P* < 0.05 for the FOXO3 targets and *P* < 0.0001 for their first-degree neighbors.

Figure S2 **Statistical comparison of gene entropy values following permutation analysis in FOXO-interaction networks.** Increasing numbers of FOXO3 targets (from 10 to 260 genes, in increment of 10 genes) and their first neighbors were randomly replaced by genes that belong to the STRING network and that are instructed by at least 6 HD-related conditions in either the mouse or human datasets, and this was performed 1000's of times. The Kolmogorov-Smirnov test was then used to compare the distributions of entropy values. Differences between distributions before and after *n* permutations (*Y* axis) are shown for *P* < 0.05 (red curves) and *P* < 0.01 (blue curves). One hundred percentage of the shuffled differences are statistically significant for replacement of less than 80–100 (mouse datasets) to 60–70 (human datasets) genes, suggesting that FOXO-interaction networks have specific entropy features. The number of permuted genes required to significantly alter the initial distributions of entropy values was greater for the mouse datasets compared to the human datasets, which reflects stronger heterogeneity in human datasets.

Figure S3 **Heat-map representing the biological content for low to high entropy genes in the FOXO3-interaction network and across seven mouse models (striatum) of HD.** Biological content is inferred from enrichment in KEGG pathways (*P* < 0.001).

Figure S4 **Heat-map representing the biological content for low to high entropy genes in the FOXO3-interaction network and across seven human HD (post-mortem caudate nucleus and cortex, blood samples from pre-and post-symptomatic HD subjects, iPS cells) datasets.** Biological content is inferred from enrichment in KEGG pathways (*P* < 0.001).

Figure S5 **Heat-map representing the biological content for low to high entropy genes in the FOXO3-interaction network and across seven mouse models of HD (striatum) and seven human HD (post-mortem caudate nucleus and cortex, blood samples from pre-and post-symptomatic HD subjects, iPS cells) datasets.** Biological content is inferred from enrichment in KEGG pathways (*P* < 0.001).

Figure S6 **Graph showing the distribution of entropy values in a KEGG pathway (here “Long-term potentiation”) that is specifically enriched in genes with high entropy values (see Figure 4)**.

## References

[B1] Anselmi C. V. Malovini A. Roncarati R. Novelli V. Villa F. Condorelli G. (2009). Association of the FOXO3A locus with extreme longevity in a southern Italian centenarian study. Rejuvenation Res. 12, 95–104 10.1089/rej.2008.0827 19415983

[B2] Bandyopadhyay S. Mehta M. Kuo D. Sung M. K. Chuang R. Jaehnig E. J. (2010). Rewiring of genetic networks in response to DNA damage. Science 330, 1385–1389 10.1126/science.1195618 21127252 PMC3006187

[B3] Borovecki F. Lovrecic L. Zhou J. Jeong H. Then F. Rosas H. D. (2005). Genome-wide expression profiling of human blood reveals biomarkers for Huntington's disease. Proc. Natl. Acad. Sci. U.S.A. 102, 11023–11028 10.1073/pnas.0504921102 16043692 PMC1182457

[B4] Burnett C. Valentini S. Cabreiro F. Goss M. Somogyvari M. Piper M. D. (2011). Absence of effects of Sir2 overexpression on lifespan in *C. elegans* and Drosophila. Nature 477, 482–485 10.1038/nature10296 21938067 PMC3188402

[B5] Califano A. (2011). Rewiring makes the difference. Mol. Syst. Biol. 7, 463 10.1038/msb.2010.117 21245848 PMC3049406

[B6] Calixto A. Jara J. S. Court F. A. (2012). Diapause formation and downregulation of insulin-like signaling via DAF-16/FOXO delays axonal degeneration and neuronal loss. PLoS Genet. 8:e1003141 10.1371/journal.pgen.1003141 23300463 PMC3531479

[B7] Calnan D. R. Brunet A. (2008). The FoxO code. Oncogene 27, 2276–2288 10.1038/onc.2008.21 18391970

[B8] Catoire H. Pasco M. Y. Abu-Baker A. Holbert S. Tourette C. Brais B. (2008). Sirtuin inhibition protects from the polyalanine muscular dystrophy protein PABPN1. Hum. Mol. Genet. 17, 2108–2117 10.1093/hmg/ddn109 18397876

[B9] Chiang W. C. Ching T. T. Lee H. C. Mousigian C. Hsu A. L. (2012). HSF-1 regulators DDL-1/2 link insulin-like signaling to heat-shock responses and modulation of longevity. Cell 148, 322–334 10.1016/j.cell.2011.12.019 22265419 PMC3615449

[B10] Christensen R. De La Torre-Ubieta L. Bonni A. Colon-Ramos D. A. (2011). A conserved PTEN/FOXO pathway regulates neuronal morphology during *C. elegans* development. Development 138, 5257–5267 10.1242/dev.069062 22069193 PMC3210501

[B11] Cohen E. Bieschke J. Perciavalle R. M. Kelly J. W. Dillin A. (2006). Opposing activities protect against age-onset proteotoxicity. Science 313, 1604–1610 10.1126/science.1124646 16902091

[B12] Cohen E. Du D. Joyce D. Kapernick E. A. Volovik Y. Kelly J. W. (2010). Temporal requirements of insulin/IGF-1 signaling for proteotoxicity protection. Aging Cell 9, 126–134 10.1111/j.1474-9726.2009.00541.x 20003171 PMC3026833

[B13] Cohen E. Paulsson J. F. Blinder P. Burstyn-Cohen T. Du D. Estepa G. (2009). Reduced IGF-1 signaling delays age-associated proteotoxicity in mice. Cell 139, 1157–1169 10.1016/j.cell.2009.11.014 20005808 PMC3017511

[B14] De La Torre-Ubieta L. Gaudilliere B. Yang Y. Ikeuchi Y. Yamada T. Dibacco S. (2010). A FOXO-Pak1 transcriptional pathway controls neuronal polarity. Genes Dev. 24, 799–813 10.1101/gad.1880510 20395366 PMC2854394

[B15] Eijkelenboom A. Burgering B. M. (2013). FOXOs: signalling integrators for homeostasis maintenance. Nat. Rev. Mol. Cell Biol. 14, 83–97 10.1038/nrm3507 23325358

[B16] HD iPSC Consortium. (2012). Induced pluripotent stem cells from patients with Huntington's disease show CAG-repeat-expansion-associated phenotypes. Cell Stem Cell 11, 264–278 10.1016/j.stem.2012.04.027 22748968 PMC3804072

[B17] Flachsbart F. Caliebe A. Kleindorp R. Blanche H. Von Eller-Eberstein H. Nikolaus S. (2009). Association of FOXO3A variation with human longevity confirmed in German centenarians. Proc. Natl. Acad. Sci. U.S.A. 106, 2700–2705 10.1073/pnas.0809594106 19196970 PMC2650329

[B18] Franceschini A. Szklarczyk D. Frankild S. Kuhn M. Simonovic M. Roth A. (2013). STRING v9.1: protein-protein interaction networks, with increased coverage and integration. Nucleic Acids Res. 41, D808–D815 10.1093/nar/gks1094 23203871 PMC3531103

[B19] Freude S. Hettich M. M. Schumann C. Stohr O. Koch L. Kohler C. (2009). Neuronal IGF-1 resistance reduces Abeta accumulation and protects against premature death in a model of Alzheimer's disease. FASEB J. 23, 3315–3324 10.1096/fj.09-132043 19487308

[B20] Fu J. Jin J. Cichewicz R. H. Hageman S. A. Ellis T. K. Xiang L. (2012). trans-(-)-epsilon-Viniferin increases mitochondrial sirtuin 3 (SIRT3), activates AMP-activated protein kinase (AMPK), and protects cells in models of Huntington Disease. J. Biol. Chem. 287, 24460–24472 10.1074/jbc.M112.382226 22648412 PMC3397871

[B21] Fuhrman S. Cunningham M. J. Wen X. Zweiger G. Seilhamer J. J. Somogyi R. (2000). The application of shannon entropy in the identification of putative drug targets. Biosystems 55, 5–14 10.1016/S0303-264700077-5 10745103

[B22] Greer E. L. Brunet A. (2005). FOXO transcription factors at the interface between longevity and tumor suppression. Oncogene 24, 7410–7425 10.1038/sj.onc.1209086 16288288

[B23] Hodges A. Strand A. D. Aragaki A. K. Kuhn A. Sengstag T. Hughes G. (2006). Regional and cellular gene expression changes in human Huntington's disease brain. Hum. Mol. Genet. 15, 965–977 10.1093/hmg/ddl013 16467349

[B24] Kanehisa M. Goto S. Kawashima S. Nakaya A. (2002). The KEGG databases at GenomeNet. Nucleic Acids Res. 30, 42–46 10.1093/nar/30.1.42 11752249 PMC99091

[B25] Kenyon C. Chang J. Gensch E. Rudner A. Tabtiang R. (1993). A *C. elegans* mutant that lives twice as long as wild type. Nature 366, 461–464 10.1038/366461a0 8247153

[B26] Killick R. Scales G. Leroy K. Causevic M. Hooper C. Irvine E. E. (2009). Deletion of Irs2 reduces amyloid deposition and rescues behavioural deficits in APP transgenic mice. Biochem. Biophys. Res. Commun. 386, 257–262 10.1016/j.bbrc.2009.06.032 19523444 PMC2726921

[B27] Kim D. Nguyen M. D. Dobbin M. M. Fischer A. Sananbenesi F. Rodgers J. T. (2007). SIRT1 deacetylase protects against neurodegeneration in models for Alzheimer's disease and amyotrophic lateral sclerosis. EMBO J. 26, 3169–3179 10.1038/sj.emboj.7601758 17581637 PMC1914106

[B28] Jeong H. Cohen D. E. Cui L. Supinski A. Savas J. N. Mazzulli J. R. (2011). Sirt1 mediates neuroprotection from mutant huntingtin by activation of the TORC1 and CREB transcriptional pathway. Nat. Med. 18, 159–165 10.1038/nm.2559 22179316 PMC3509213

[B29] Jiang M. Wang J. Fu J. Du L. Jeong H. West T. (2011). Neuroprotective role of Sirt1 in mammalian models of Huntington's disease through activation of multiple Sirt1 targets. Nat. Med. 18, 153–158 10.1038/nm.2558 22179319 PMC4551453

[B30] Kleindorp R. Flachsbart F. Puca A. A. Malovini A. Schreiber S. Nebel A. (2011). Candidate gene study of FOXO1, FOXO4, and FOXO6 reveals no association with human longevity in Germans. Aging Cell 10, 622–628 10.1111/j.1474-9726.2011.00698.x 21388494

[B31] Kuhn A. Goldstein D. R. Hodges A. Strand A. D. Sengstag T. Kooperberg C. (2007). Mutant huntingtin's effects on striatal gene expression in mice recapitulate changes observed in human Huntington's disease brain and do not differ with mutant huntingtin length or wild-type huntingtin dosage. Hum. Mol. Genet. 16, 1845–1861 10.1093/hmg/ddm133 17519223

[B32] Landis J. N. Murphy C. T. (2010). Integration of diverse inputs in the regulation of Caenorhabditis elegans DAF-16/FOXO. Dev. Dyn. 239, 1405–1412 10.1002/dvdy.22244 20140911 PMC3811053

[B33] Lefebvre C. Rieckhof G. Califano A. (2012). Reverse-enginering human regulatory networks. Wiley Interdiscip. Rev. Syst. Biol. Med. 4, 311–325 10.1002/wsbm.1159 22246697 PMC4128340

[B34] Lejeune F. X. Mesrob L. Parmentier F. Bicep C. Vazquez-Manrique R. P. Parker J. A. (2012). Large-scale functional RNAi screen in *C. elegans* identifies genes that regulate the dysfunction of mutant polyglutamine neurons. BMC Genomics 13:91 10.1186/1471-2164-13-91 22413862 PMC3331833

[B35] Li Y. Wang W. J. Cao H. Lu J. Wu C. Hu F. Y. (2009). Genetic association of FOXO1A and FOXO3A with longevity trait in Han Chinese populations. Hum. Mol. Genet. 18, 4897–4904 10.1093/hmg/ddp459 19793722 PMC2790334

[B36] Mielke P. W. Jr. Berry K. J. (2007). Permutation Methods: a Distance Function Approach. New York, NY: Springer

[B37] Mei Y. Wang Z. Zhang L. Zhang Y. Li X. Liu H. (2012). Regulation of neuroblastoma differentiation by forkhead transcription factors FOXO1/3/4 through the receptor tyrosine kinase PDGFRA. Proc. Natl. Acad. Sci. U.S.A. 109, 4898–4903 10.1073/pnas.1119535109 22411791 PMC3323967

[B38] Morley J. F. Brignull H. R. Weyers J. J. Morimoto R. I. (2002). The threshold for polyglutamine-expansion protein aggregation and cellular toxicity is dynamic and influenced by aging in Caenorhabditiselegans. Proc. Natl. Acad. Sci. U.S.A. 16, 16 10.1073/pnas.152161099 12122205 PMC124929

[B39] Neri C. (2012). Role and therapeutic potential of the pro-longevity factor FOXO and its regulators in neurodegenerative disease. Front. Pharmacol. 3:15 10.3389/fphar.2012.00015 22363285 PMC3281233

[B40] Orth M. Handley O. J. Schwenke C. Dunnett S. Wild E. J. Tabrizi S. J. (2011). Observing Huntington's disease: the European Huntington's disease network's REGISTRY. J. Neurol. Neurosurg. Psychiatry 82, 1409–1412 10.1136/jnnp.2010.209668 21097549

[B41] Paik J. H. Ding Z. Narurkar R. Ramkissoon S. Muller F. Kamoun W. S. (2009). FoxOs cooperatively regulate diverse pathways governing neural stem cell homeostasis. Cell Stem Cell 5, 540–553 10.1016/j.stem.2009.09.013 19896444 PMC3285492

[B42] Parker J. A. Arango M. Abderrahmane S. Lambert E. Tourette C. Catoire H. (2005). Resveratrol rescues mutant polyglutamine cytotoxicity in nematode and mammalian neurons. Nat. Genet. 37, 349–350 10.1038/ng1534 15793589

[B43] Parker J. A. Vazquez-Manrique R. P. Tourette C. Farina F. Offner N. Mukhopadhyay A. (2012). Integration of beta-catenin, sirtuin, and FOXO signaling protects from mutant huntingtin toxicity. J. Neurosci. 32, 12630–12640 10.1523/JNEUROSCI.0277-12.2012 22956852 PMC3780431

[B44] Pasco M. Y. Catoire H. Parker J. A. Brais B. Rouleau G. A. Neri C. (2010). Cross-talk between canonical Wnt signaling and the sirtuin-FoxO longevity pathway to protect against muscular pathology induced by mutant PABPN1 expression in *C. elegans*. Neurobiol. Dis. 38, 425–433 10.1016/j.nbd.2010.03.002 20227501

[B45] Rapaport F. Zinovyev A. Dutreix M. Barillot E. Vert J. P. (2007). Classification of microarray data using gene networks. BMC Bioinformatics 8:35 10.1186/1471-2105-8-35 17270037 PMC1797191

[B46] Renault V. M. Rafalski V. A. Morgan A. A. Salih D. A. Brett J. O. Webb A. E. (2009). FoxO3 regulates neural stem cell homeostasis. Cell Stem Cell 5, 527–539 10.1016/j.stem.2009.09.014 19896443 PMC2775802

[B47] Ro S. H. Liu D. Yeo H. Paik J. H. (2012). FoxOs in neural stem cell fate decision. Arch. Biochem. Biophys. 534, 55–63 10.1016/j.abb.2012.07.017 22902436

[B48] Russ A. P. Lampel S. (2005). The druggable genome: an update. Drug Discov. Today 10, 1607–1610 10.1016/S1359-6446(05)03666-4 16376820

[B49] Salih D. A. Rashid A. J. Colas D. De La Torre-Ubieta L. Zhu R. P. Morgan A. A. (2012). FoxO6 regulates memory consolidation and synaptic function. Genes Dev. 26, 2780–2801 10.1101/gad.208926.112 23222102 PMC3533081

[B50] Shou C. Bhardwaj N. Lam H. Y. Yan K. K. Kim P. M. Snyder M. (2011). Measuring the evolutionary rewiring of biological networks. PLoS Comput. Biol. 7:e1001050 10.1371/journal.pcbi.1001050 21253555 PMC3017101

[B51] Soerensen M. Dato S. Christensen K. McGue M. Stevnsner T. Bohr V. A. (2010). Replication of an association of variation in the FOXO3A gene with human longevity using both case-control and longitudinal data. Aging Cell 9, 1010–1017 10.1111/j.1474-9726.2010.00627.x 20849522 PMC2992870

[B52] Tang H. Y. Smith-Caldas M. S. Driscoll M. V. Salhadar S. Shingleton A. W. (2011). FOXO regulates organ-specific phenotypic plasticity in Drosophila. PLoS Genet. 7:e1002373 10.1371/journal.pgen.1002373 22102829 PMC3213149

[B53] Teixeira-Castro A. Ailion M. Jalles A. Brignull H. R. Vilaca J. L. Dias N. (2011). Neuron-specific proteotoxicity of mutant ataxin-3 in *C. elegans*: rescue by the DAF-16 and HSF-1 pathways. Hum. Mol. Genet. 20, 2996–3009 10.1093/hmg/ddr203 21546381 PMC3131043

[B54] Thomas E. A. Coppola G. Tang B. Kuhn A. Kim S. Geschwind D. H. (2011). *In vivo* cell-autonomous transcriptional abnormalities revealed in mice expressing mutant huntingtin in striatal but not cortical neurons. Hum. Mol. Genet. 20, 1049–1060 10.1093/hmg/ddq548 21177255 PMC3043657

[B55] Walker F. O. (2007). Huntington's disease. Lancet 369, 218–228 10.1016/S0140-6736(07)60111-1 17240289

[B56] West J. Bianconi G. Severini S. Teschendorff A. E. (2012). Differential network entropy reveals cancer system hallmarks. Sci. Rep. 2, 802 10.1038/srep00802 23150773 PMC3496163

[B57] Willcox B. J. Donlon T. A. He Q. Chen R. Grove J. S. Yano K. (2008). FOXO3A genotype is strongly associated with human longevity. Proc. Natl. Acad. Sci. U.S.A. 105, 13987–13992 10.1073/pnas.0801030105 18765803 PMC2544566

[B58] Yen K. Narasimhan S. D. Tissenbaum H. A. (2011). DAF-16/Forkhead box O transcription factor: many paths to a single Fork(head) in the road. Antioxid. Redox. Signal. 14, 623–634 10.1089/ars.2010.3490 20673162 PMC3021330

[B59] Zhang T. Mullane P. C. Periz G. Wang J. (2011). TDP-43 neurotoxicity and protein aggregation modulated by heat shock factor and insulin/IGF-1 signaling. Hum. Mol. Genet. 20, 1952–1965 10.1093/hmg/ddr076 21355045 PMC3080607

[B60] Zuccato C. Valenza M. Cattaneo E. (2010). Molecular mechanisms and potential therapeutical targets in Huntington's disease. Physiol. Rev. 90, 905–981 10.1152/physrev.00041.2009 20664076

