# Supplementary material for: Pathways to decoding the clinical potential of stress response FOXO-interaction networks for Huntington's disease: of gene prioritization and context dependence
Source: Front Aging Neurosci. 2013 Jun 13;5:22. doi: 10.3389/fnagi.2013.00022 (PMC3680703; doi:10.3389/fnagi.2013.00022)
Supplement: Table S1 — Fourteen models of HD pathogenesis for which transcriptomic data are available. [file DataSheet1.ZIP › Supplementary Material - Pathways to decoding the clinical potential of stress response FOXO-interaction networks for Huntingtons disease of gene prioritization and context dependence/50245__Table_1.docx]

**Table S1.** Fourteen models of HD pathogenesis for which transcriptomic data are available.

| **Model** | **Species** | **(CAG)n** | **Origin** | **Number of samples (HD/normal)** | **Age-at-onset**  **of HD (years)** | **Pathological stage or grade** | **Reference** |
| --- | --- | --- | --- | --- | --- | --- | --- |
| Transgenic R6/2 | Mouse | 150-209 | Striatum | 7 / 7 | Na | Early stage  (6 weeks) | Kuhn *et al*, Hum Mol Genet 2007 |
| Transgenic R6/2 |  |  |  | 5 / 4 | Na | Late stage  (12 weeks) |  |
| Knock-in CHL2 |  | 150 |  | 4 / 4 | Na | Late stage  (22 months) |  |
| Transgenic YAC128 |  | 128 |  | 4 / 4 | Na | Early stage  (12 months) |  |
| Transgenic YAC128 |  | 128 |  | 6 / 4 | Na | Late stage  (24 months) |  |
| Knock-in Q92 |  | 92 |  | 3 / 3 | Na | Late stage  (18 months) |  |
| Transgenic DE5 |  | 98 |  | 4 / 4 | Na | Late stage  (14 months) | Thomas *et al*, Hum Mol Genet 2011 |
| Post-mortem brain | Human | 43* | Cortex BA4 | 44 / 36 | 58* | Grade 0-4 | Hodges *et al*, Hum Mol Genet 2006 |
|  |  |  | Cortex BA9 |  |  |  |  |
|  |  |  | Caudate nucleus |  |  |  |  |
| Blood |  | NA | pre-symptomatic HD | 5 | 39* | Na | Borovecki *et al*, PNAS 2005 |
|  |  | NA | manifest HD | 12 / 14 | 49.6* | Grade 1-2 |  |
| Neural stem cell |  | 60 | Induced pluripotent stem cell | 2 / 3 | 18 | Na | The HD iPSC Consortium, Cell Stem Cell 2012 |
|  |  | 180 |  | 2 / 3 | < 6 | Na |  |

NA: not available

Na: not applicable

*mean value
