# Supplementary material for: Pathways to decoding the clinical potential of stress response FOXO-interaction networks for Huntington's disease: of gene prioritization and context dependence
Source: Front Aging Neurosci. 2013 Jun 13;5:22. doi: 10.3389/fnagi.2013.00022 (PMC3680703; doi:10.3389/fnagi.2013.00022)
Supplement: Table S1 — Fourteen models of HD pathogenesis for which transcriptomic data are available. [file DataSheet1.ZIP › Supplementary Material - Pathways to decoding the clinical potential of stress response FOXO-interaction networks for Huntingtons disease of gene prioritization and context dependence/Supplementary Figures.pdf]

Supplementary figures

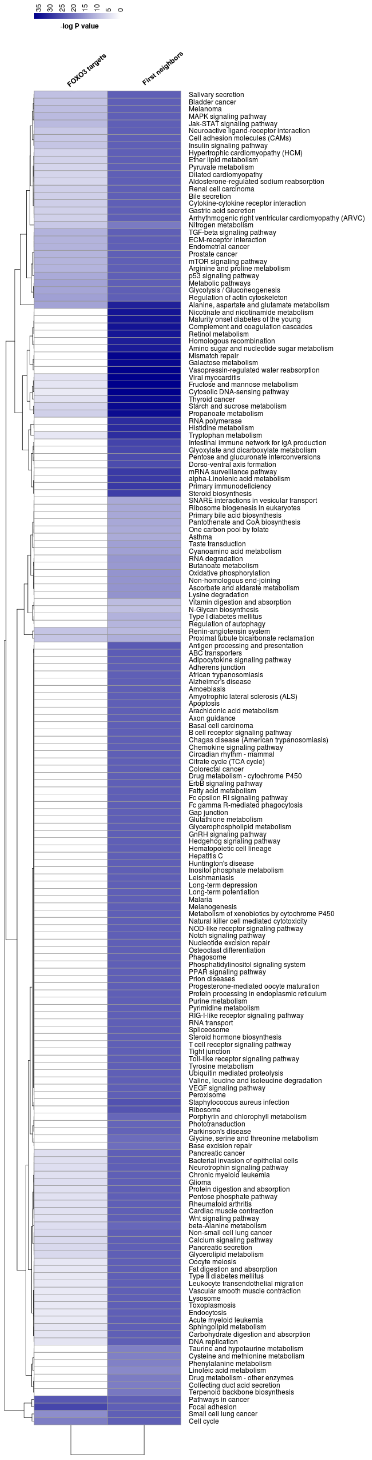

Figure S1. Heat-map representing the biological content for 350 mouse FOXO3 targets and their 5859 first neighbors (STRING confidence score greater than 0.4). Biological annotations are inferred from KEGG pathways. The cut-off for enrichment in KEGG pathways is $P < 0.05$ for the FOXO3 targets and $P < 0.0001$ for their first-degree neighbors.

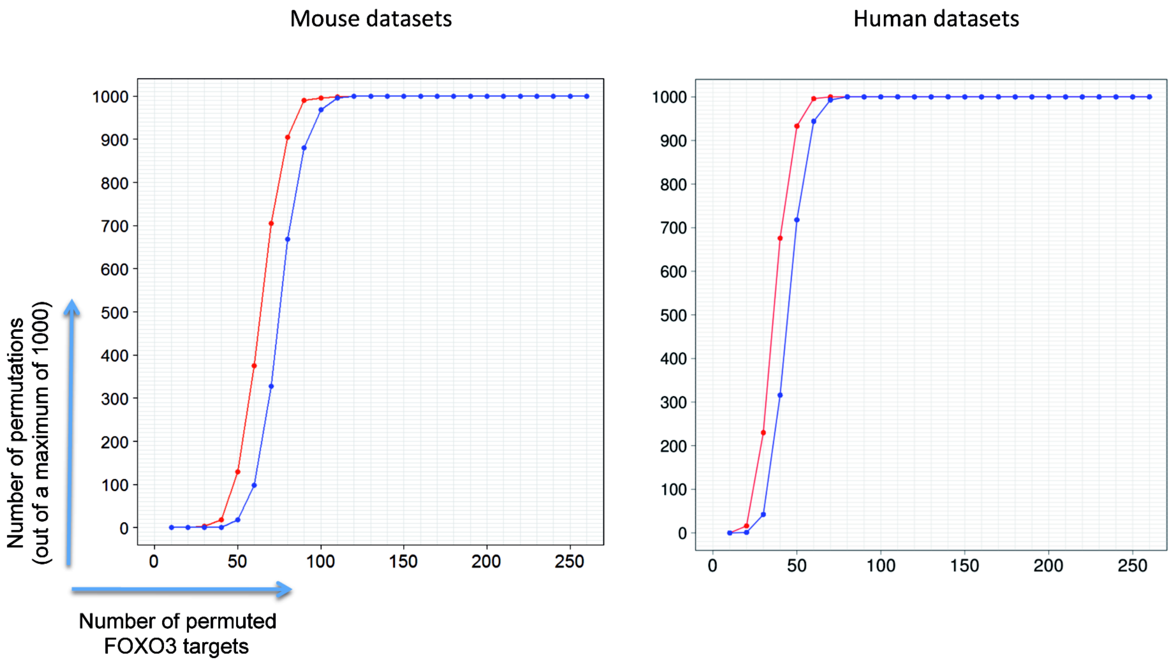

Figure S2. Statistical comparison of gene entropy values following permutation analysis in FOXO-interaction networks. Increasing numbers of FOXO3 targets (from 10 to 260 genes, in increment of 10 genes) and their first neighbors were randomly replaced by genes that belong to the STRING network and that are instructed by at least 6 HD-related conditions in either the mouse or human datasets, and this was performed 1000's of times. The Kolmogorov-Smirnov test was then used to compare the distributions of entropy values. Differences between distributions before and after n permutations (Y axis) are shown for $P < 0.05$ (red curves) and $P < 0.01$ (blue curves). 100% of the shuffled differences are statistically significant for replacement of less than 80-100 (mouse datasets) to 60-70 (human datasets) genes, suggesting that FOXO-interaction networks have specific entropy features. The number of permuted genes required to significantly alter the initial distributions of entropy values was greater for the mouse datasets compared to the human datasets, which reflects stronger heterogeneity in human datasets.

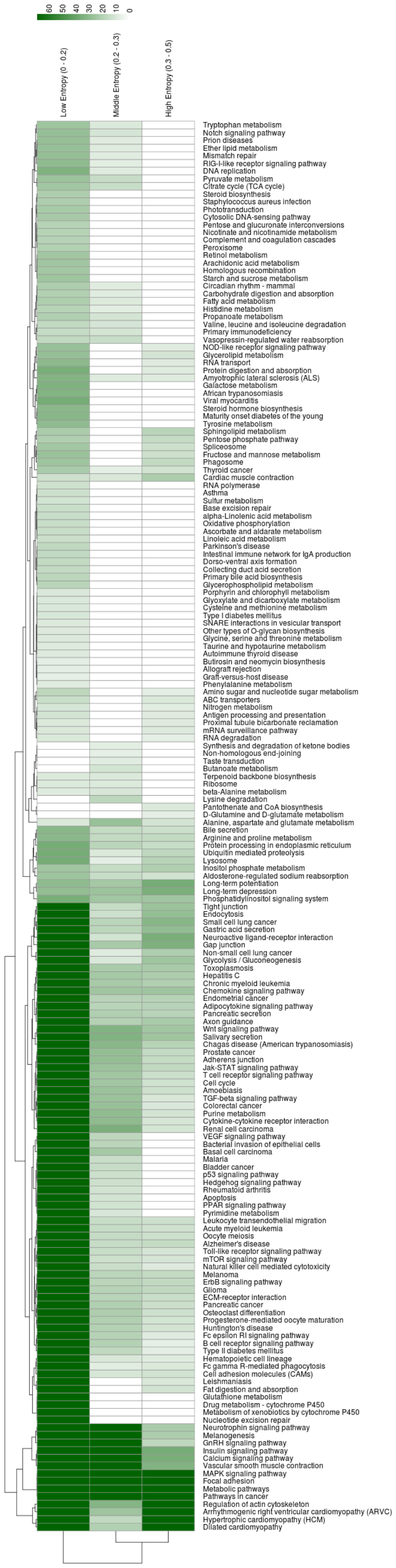

Figure S3. Heat-map representing the biological content for low to high entropy genes in the FOXO3-interaction network and across seven mouse models (striatum) of HD. Biological content is inferred from enrichment in KEGG pathways ($P < 0.001$).

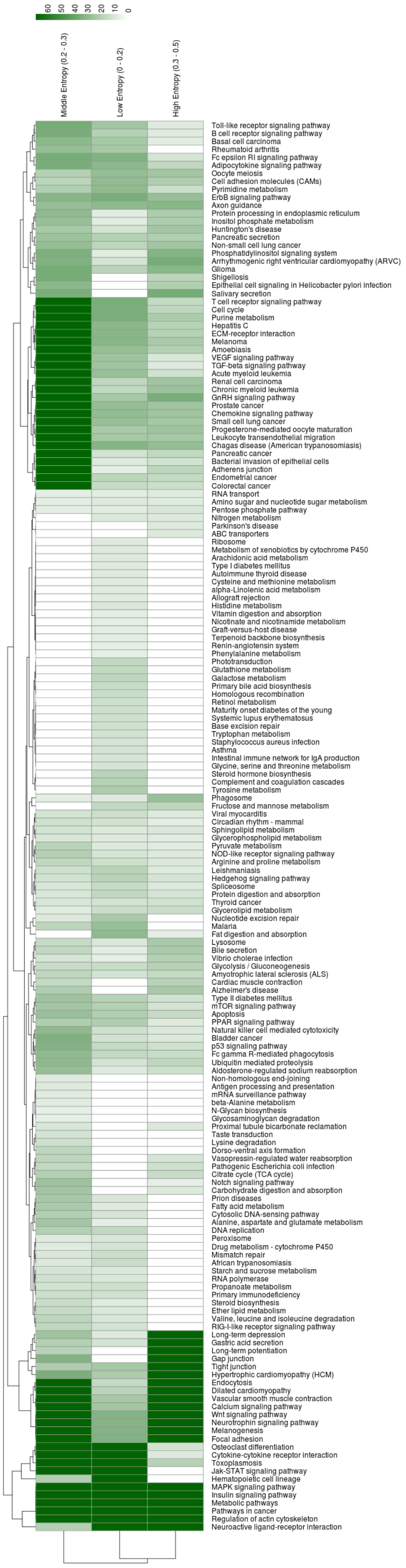

Figure S4. Heat-map representing the biological content for low to high entropy genes in the FOXO3-interaction network and across seven human HD (post-mortem caudate nucleus and cortex, blood samples from pre-and post-symptomatic HD subjects, iPS cells) datasets. Biological content is inferred from enrichment in KEGG pathways ($P < 0.001$).

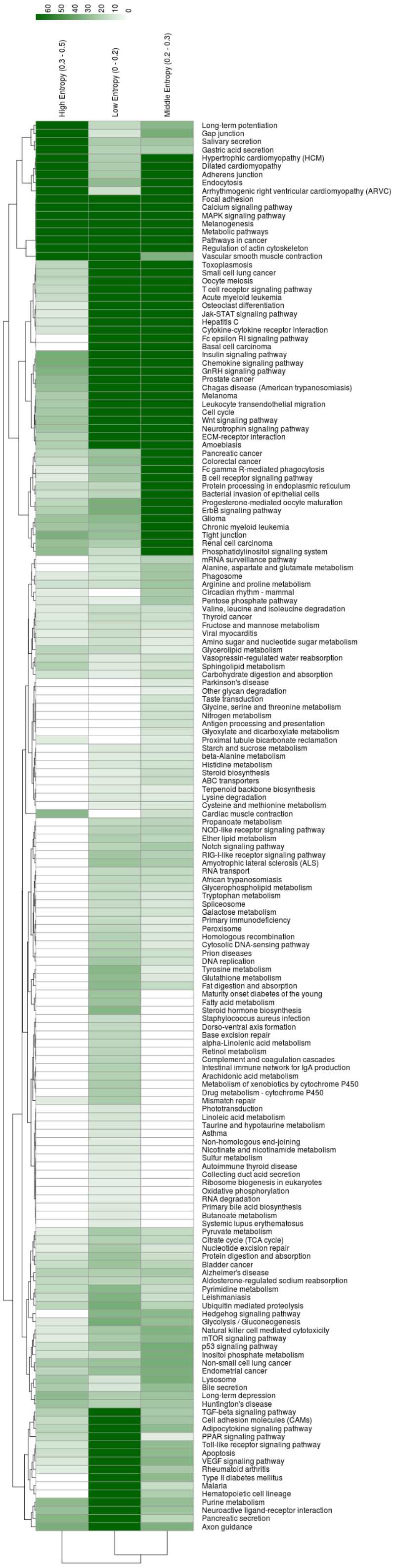

Figure S5. Heat-map representing the biological content for low to high entropy genes in the FOXO3-interaction network and across seven mouse models of HD (striatum) and seven human HD (post-mortem caudate nucleus and cortex, blood samples from pre-and post-symptomatic HD subjects, iPS cells) datasets. Biological content is inferred from enrichment in KEGG pathways ($P < 0.001$).

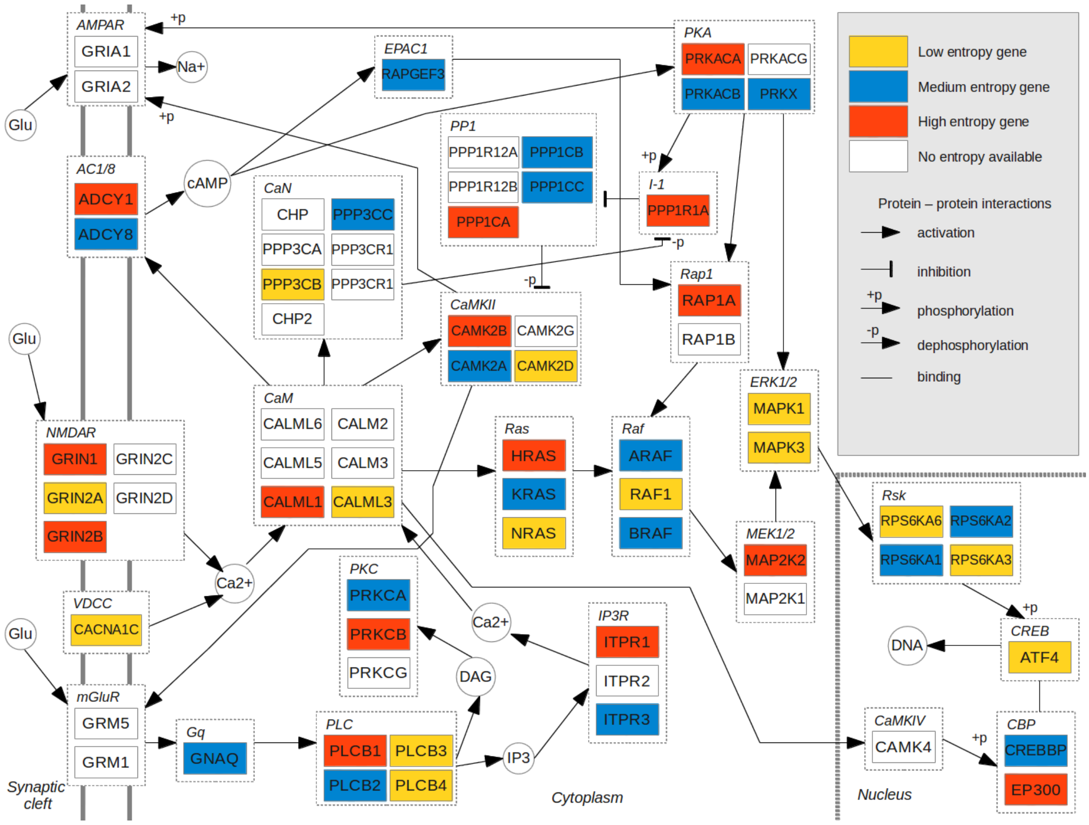

Figure S6. Graph showing the distribution of entropy values in a KEGG pathway (here 'Long-term potentiation') that is specifically enriched in genes with high entropy values (see Fig. 4).
